# Integrating Temporal and Spatial Dimensions of Alpine Adaptation: Camera‐Trap Insights on Bharal ( *Pseudois nayaur* ) in Giant Panda National Park

**DOI:** 10.1002/ece3.71874

**Published:** 2025-07-28

**Authors:** Rumei Zhang, Chen Yang, Ding Zhao, Dehong Pang, Weichao Zheng, Tianpei Guan, Zhuo Tang

**Affiliations:** ^1^ College of Grassland Resources Southwest Minzu University Chengdu China; ^2^ School of Environment Beijing Normal University Beijing China; ^3^ Sichuan Xuebaoding National Nature Reserve Mianyang China; ^4^ Sichuan Academy of Giant Panda Chengdu China; ^5^ Administration Bureau of Wolong National Nature Reserve Wenchuan China

**Keywords:** activity strategies, alpine ungulates, Giant Panda National Park, infrared camera

## Abstract

Alpine ungulates exemplify climate vulnerability through their spatiotemporal adaptation strategies, yet integrated analyses of these dimensions remain scarce. Here, we investigated how bharal ( *Pseudois nayaur* ) in Giant Panda National Park adjusts both time‐activity budgets and spatial distributions under extreme seasonal conditions. We deployed a network of 50 infrared cameras along altitudinal transects (3300–4500 m) during summer and winter. We extracted the Normalized Difference Vegetation Index (NDVI) from satellite imagery for each camera site and calculated the Relative Abundance Index (RAI) to quantify activity intensity and assess its seasonal variation. Our results revealed two key adaptations. (i) Temporal compression: Activity intensity in winter was reduced by 66% compared to summer (RAI: 0.85 ± 0.04 vs. 0.29 ± 0.21; *p* < 0.01) and exhibited a weaker diurnal‐nocturnal contrast (*p* < 0.05). (ii) Spatial contraction: The bharal's altitudinal range narrowed by 73% from summer (3685–4248 m) to winter (3859–4012 m), accompanied by a significant decrease in NDVI (summer: 0.70 ± 0.14 vs. winter: 0.14 ± 0.06; *p* < 0.05). These findings reveal a dual‐phase adaptation in bharal: expanding activity and range in resource‐rich summers and contracting both in winter to conserve energy. By integrating infrared camera data with satellite‐derived NDVI, our approach highlights how alpine ungulates respond to seasonal challenges and provides a foundation for predicting climate‐driven shifts in high‐elevation ecosystems.

## Graphical abstract

This study integrates infrared camera‐trap data to reveal the spatiotemporal adaptation strategies of bharal (*Pseudois nayaur*) in China's Giant Panda National Park. We found a 66% reduction in winter activity intensity and a 73% contraction in altitudinal range, demonstrating a dual‐phase strategy of temporal compression and spatial contraction to optimize energy use under extreme alpine conditions. These insights advance climate adaptation research for alpine ungulates and inform conservation strategies for high‐elevation ecosystems.

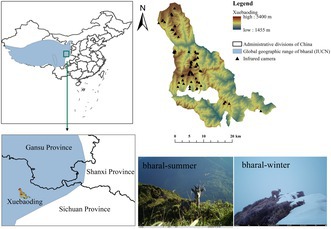

## 1 Introduction

Alpine environments are characterized by extreme climatic conditions, including low oxygen availability (often below 60% of sea‐level values), severe temperature fluctuations (with diurnal variations exceeding 30°C), limited nutritional resources (Grignolio et al. 2018; Seigle‐Ferrand et al. 2022), and pronounced seasonal variability (Kohli et al. 2014). These harsh conditions impose significant ecological challenges for wildlife inhabiting alpine ecosystems, driving them to develop specialized adaptations that enable survival and reproduction (Bears et al. 2009; Brivio et al. 2016). Understanding how wildlife species respond and adapt to these environmental pressures is essential for biodiversity conservation and effective management of alpine habitats.

Adaptation in wildlife can be broadly categorized into temporal and spatial dimensions (Gervasi et al. 2014; Karanth et al. 2017). On the one hand, temporal adaptation refers to behavioral and physiological changes occurring over time (Teitelbaum et al. 2018), such as seasonal adjustments in activity patterns, reproductive strategies, or dietary preferences (Vazquez et al. 2019; Johansson et al. 2022). Spatial adaptation, on the other hand, involves variations in habitat use and distribution across different geographic or altitudinal gradients, reflecting responses to resource availability, predation risk, and environmental conditions (Wu et al. 2017; Lamb et al. 2025). Investigating both temporal and spatial adaptations provides comprehensive insights into the strategies species employ to cope with complex alpine environments.

The bharal (blue sheep, *Pseudois nayaur* ) serves as an ideal subject for studying alpine adaptation due to its wide distribution in high‐altitude regions across the Tibetan Plateau and adjacent mountain ranges (Wang et al. 2024). Bharal populations exhibit remarkable abilities to navigate steep terrain, utilize sparse vegetation, and withstand extreme weather conditions (Marschall 1998; Xiao et al. 2022), making them particularly suitable for examining adaptive responses to alpine stressors. Despite their ecological significance (Espunyes et al. 2019; Fluri et al. 2023), detailed studies on bharal's adaptive strategies, especially concerning temporal and spatial dynamics, remain limited.

Giant Panda National Park, located in southwestern China, encompasses diverse ecosystems across a substantial altitudinal gradient (1100–5400 m above sea level) (Li et al. 2024). Although primarily established for giant panda conservation, the park's extensive alpine habitats support a rich diversity of wildlife, including prominent populations of bharal (Li et al. 2021; Zhang et al. 2023). The park's varied topography, ranging from temperate forests to alpine meadows, presents an ideal natural laboratory for investigating wildlife adaptation (Zhang et al. 2023). Furthermore, ongoing ecological monitoring programs, including camera traps, provide valuable data resources for comprehensive wildlife studies (Tian et al. 2021).

Currently, there is a notable knowledge gap regarding how bharal temporally and spatially adapt to alpine environments within Giant Panda National Park. Previous studies have rarely integrated both temporal and spatial dimensions of adaptation, leaving critical questions unanswered about bharal habitat use and behavioral adjustments across different environmental contexts (Wang et al. 2024; Fu et al. 2025). Studies have shown that NDVI and temperature are significantly associated with habitat suitability for ungulates and serve as important indicators of changes in their activity patterns (Liu et al. 2015; Ito et al. 2018). To quantify key environmental drivers and elucidate the ecological basis of bharal adaptive strategies, we integrated NDVI and temperature as core environmental variables in this study. Specifically, we aimed to investigate the temporal and spatial patterns of bharal habitat use and behavior within Giant Panda National Park, focusing on how these animals adapt to seasonal and altitudinal variations in their alpine habitats. By combining camera‐trap data on activity patterns with environmental variables, we sought to elucidate the adaptive strategies employed by bharal in response to environmental pressures.

Understanding bharal adaptation mechanisms has significant implications for alpine ecology, conservation biology, and protected area management (Yang et al. 2023; Luo et al. 2024; Orazi et al. 2024). Insights gained from this research will contribute to improved conservation strategies for bharal and other alpine species, inform habitat management practices, and enhance our broader understanding of wildlife responses to environmental variability. We hypothesized that bharal exhibit distinct temporal and spatial adaptations, including seasonal shifts in habitat selection and altitudinal migrations, enabling them to optimize resource availability and minimize exposure to alpine environmental extremes.

## 2 Materials and Methods

### 2.1 Study Area

This study was conducted in Xuebaoding (coordinates 103°50′–104°18′ E, 32°14′–32°38′ N), situated in the northwestern region of Pingwu county, Sichuan province, China, covering an area of 1099 km^2^. The altitude extends from 1350 m to 5400 m, giving rise to a distinct vertical vegetation gradient. Vegetation transitions occur from deciduous broad‐leaved forest, mixed coniferous forest, and coniferous forest, followed by alpine scrub and scrub meadow between 3300 m and 4000 m, and ultimately alpine meadow and screes above 4000 m (Ji et al. 2021). Xuebaoding is widely recognized as a critical element of the global biodiversity hotspots in southwest China, playing an indispensable role in conserving the population and habitat of emblematic species such as giant panda ( *Ailuropoda melanoleuca* ), takin ( *Budorcas taxicolor* ), Sichuan snub‐nosed monkey ( *Rhinopithecus roxellana* ) and Chinese dove tree (*Davidia involucrata*) (Zhao et al. 2021).

### 2.2 Data Collection

**FIGURE 1:**
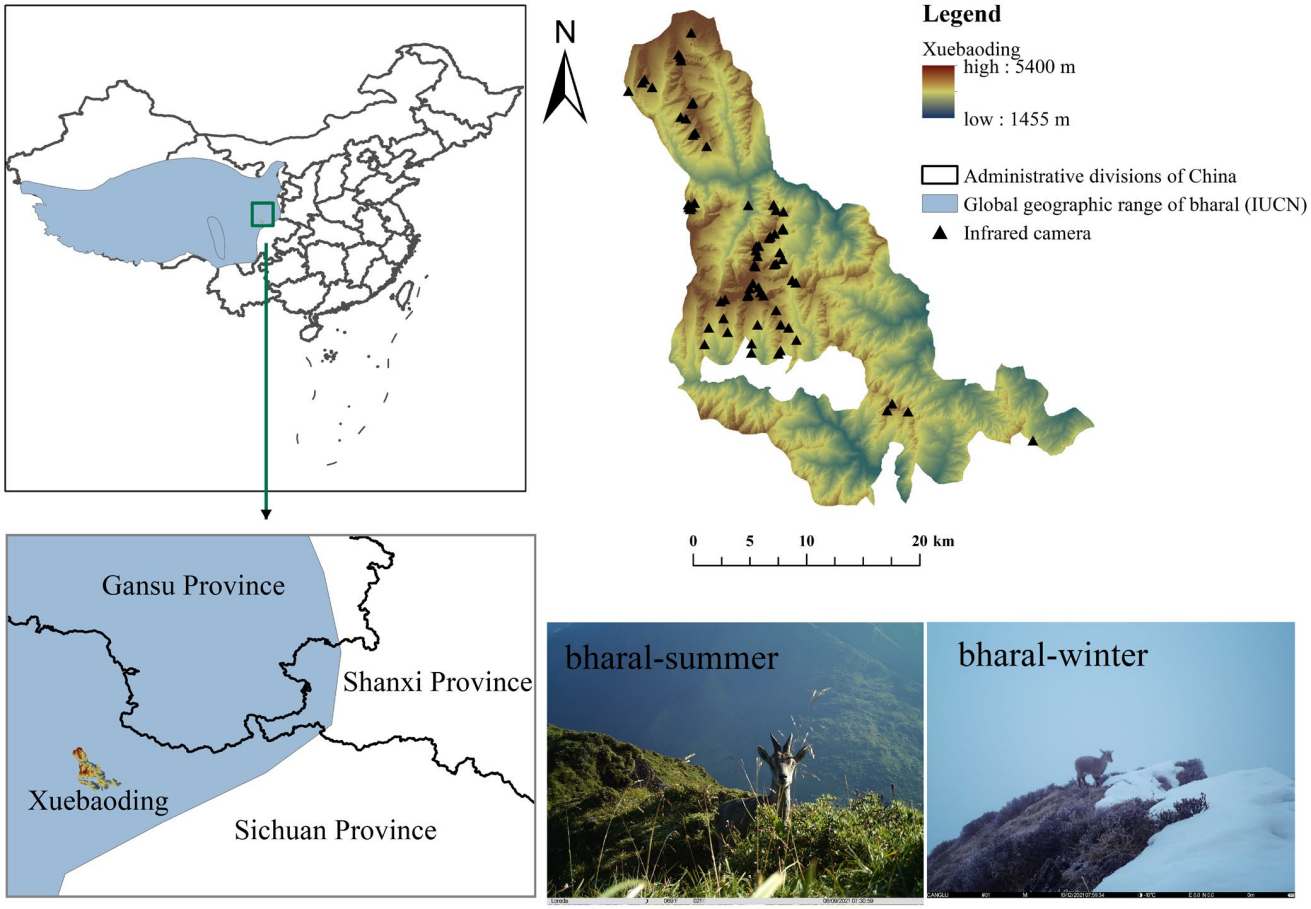
Infrared camera location in Xuebaoding, Giant Panda National Park.

From June 2020 to July 2022, a total of 50 infrared cameras were deployed along an elevation gradient from 3300 to 4500 m in alpine meadow habitats, based on wildlife signs and accessibility (Figure 1). Specifically, 26 cameras were installed at elevations between 3300 and 4000 m, and 24 were placed between 4000 and 4500 m. The specific type of camera used was yianws‐L710. The camera parameters were set as follows: photo resolution = 1080 P, video length = 10 s, trigger interval = 1 min, three photos, and using 24‐h time format. The cameras were securely fixed on the tree trunks at a height ranging from 0.5 to 1 m along the treeline or on a hand‐built rockpile in the meadow. Data collection occurred every 3 or 4 months. Detailed information regarding camera setup protocols and deployment methods can be found in existing literature (Li et al. 2014).

We investigated the influence of normalized difference vegetation index (NDVI), a reliable satellite‐based method for monitoring surface vegetation dynamics that is closely associated with vegetation coverage (Linderholm 2006). The NDVI data used in this study were derived from monthly vegetation indices in summer and winter with a spatial resolution of 1 km (https://www.resdc.cn/Default.aspx, 2020). The ambient temperature data were collected by infrared camera trap (Zhou et al. 2022).

### 2.3 Defining the Processing of Infrared Camera Data

Initially, a continuous 24‐h operation of a single infrared camera was considered as a camera day. Relevant information including camera day, photo date, and shooting time was automatically extracted by using Bio‐photo V2.1 to generate a data sheet (Liu et al. 2023). Subsequently, trained operators screened each image and video clip to identify recorded species within each trigger. Records pertaining to bharal were then extracted and analyzed accordingly. The relative abundance index (RAI) was used as the basis for quantifying species activity intensity (Li et al. 2014):

$$\mathrm{RAI}=100\times \frac{\text{number of independent photos}}{\text{total effective camera days}}$$

An independent detection of a species was defined as all photographs of that species at a single sampling site separated by an interval of more than 30 min (Li et al. 2012). We adjusted the time interval from 30 to 10 min to identify independent photos (IP). This is the first time to attempt to reduce the ignored activity intensity by shortening the time interval of independent photos during the calculation process. In this study, consecutive photos of the same species taken within 10 min were considered a single independent event and referred to as one IP.

### 2.4 Data Analysis

In spatial analysis, we initially calculated the RAI of bharal at each camera site during both summer and winter. Subsequently, the study area (3300–4500 m) was divided into two altitude bands based on vegetation type: a low altitude band (3300–4000 m) with a total of 26 cameras, and a high altitude band (4000–4500 m) with a total of 24 cameras. The RAI of bharal in these two altitude bands was compared to assess variations in activity patterns across seasons and altitudes.

In temporal analysis, we utilized local climate data, infrared camera images, and seasonal classification in the literature (Hazlerigg and Tyler 2019; Farsi et al. 2022) to classify June to August as summer and December to February as winter. The average time of sunrise and sunset was employed as the criterion for distinguishing between day and night within each season (e.g., summer daytime from 06:00 to 20:00, nighttime from 20:00 to 06:00 the next day; winter daytime from 08:00 to 18:00, nighttime from 18:00 to 08:00 the next day) (Farsi et al. 2022). The activity intensity of bharal was calculated according to the number of IPs captured during different seasons and periods of either days or nights. The RAI was expressed as mean ± standard deviation (Mean ± SD). Before conducting the comparative analysis, data normality was assessed to determine the appropriate statistical test. If the data followed a normal distribution, analysis of variance was used. For non‐normal distributions, non‐parametric tests were applied (Wilcoxon signed‐rank or Kruskal–Wallis). A significance level of *p* < 0.05 was set for all tests.

We employed Kernel density estimation to compare the diurnal activity patterns of bharal between seasons, assuming that their behavioral activities were continuously distributed within a 24‐h cycle. IPs were considered as random samples from this distribution (Ridout and Linkie 2009). Firstly, we utilized the densityPlot function (R 4.2.2) from the overlap package to generate single‐season kernel density curves, illustrating the activity patterns. The overlapPlot function was then applied to visualize their daily activity rhythms during summer and winter. Secondly, we selected overlap coefficients based on smaller sample sizes between different seasons (Dhat 1 is suitable for sample size < 50; Dhat 4 is suitable for sample size > 75), with results expressed as a 0–1 scale indicating the degree of overlap (0 indicates complete separation, 1 indicates complete overlap). Lastly, the compareCkern function in the activity package was used to assess the likelihood of seasonal difference in bharal daily activity rhythms through 1000 permutation cycles (Azevedo et al. 2018; Chen et al. 2019).

## 3 Results

Our monitoring covered 4050 camera days for summer and 3985 camera days for winter. In total, 1156 IPs of bharal were captured in summer and 108 IPs were captured in the winter. Cameras positioned at higher altitudes operated for a duration of 2435 days during summer and 1339 days during winter, capturing a total 1001 IPs in summer datasets and 84 IPs in winter datasets. Cameras placed at lower altitudes remained active for a duration of 2266 days during summer and 1995 days during winter, capturing 155 IPs in summer records and merely 24 IPs from winter cameras.

### 3.1 Environmental Characteristics

**FIGURE 2:**
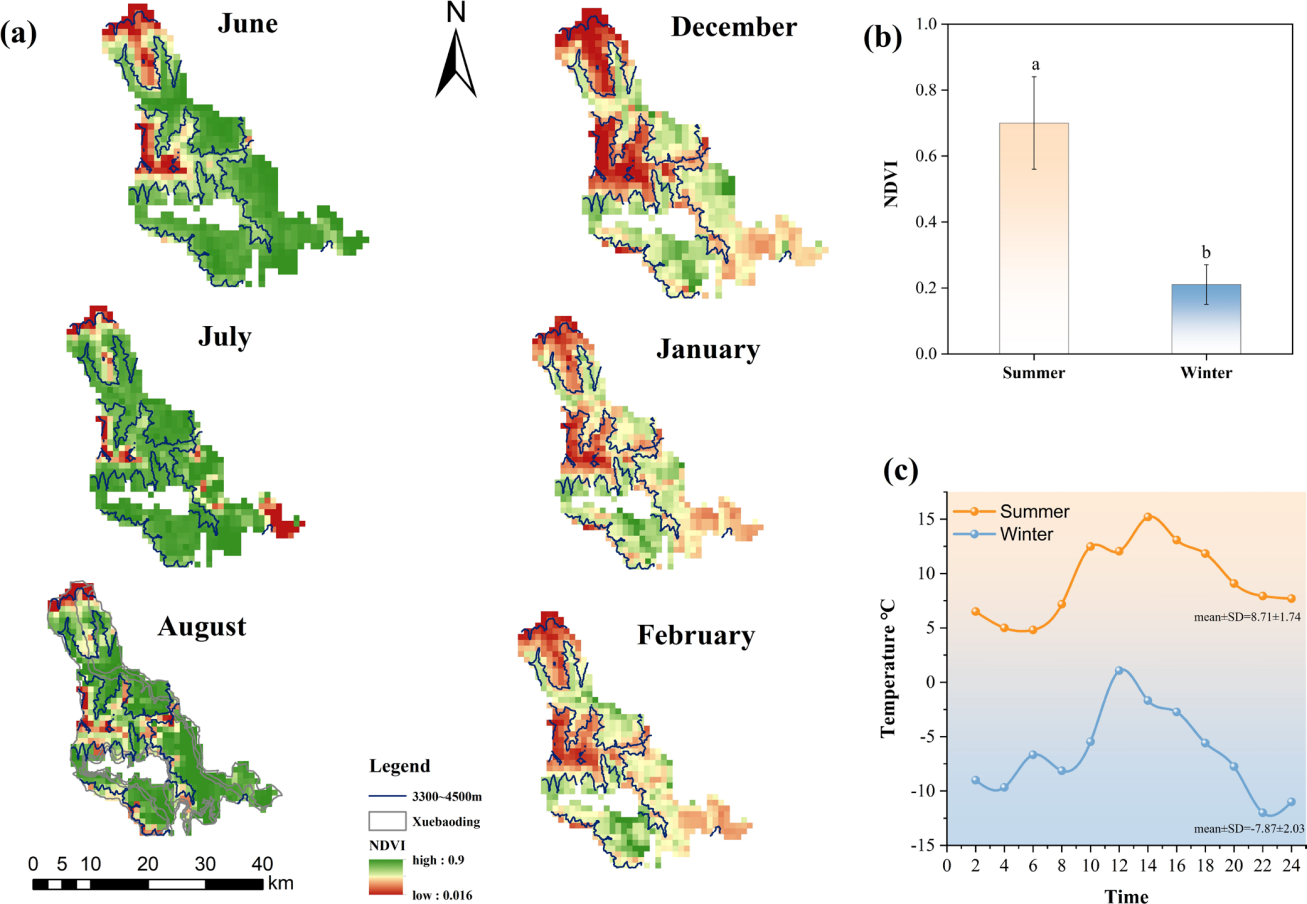
Seasonal differences in the environmental characteristics. Caption (a) is the monthly NDVI at Xuebaoding in summer and winter. The blue contour line represents the surveyed area (3300–4500 m). (b) The seasonal difference in mean daily temperatures at bharal activity sites. (c) The seasonal difference in NDVI at the bharal activity site.

NDVI in 2020 varied among months (Figure 2a). There was a significant increase in NDVI at the bharal distribution sites during summer (NDVI = 0.7 ± 0.14), compared to winter (NDVI = 0.14 ± 0.06, *p* < 0.05) (Figure 2b). During summer, the average daily temperature at the activity sites of bharal reached 8.7°C, with a maximum temperature of 15.2°C at 14:00. During winter, the average daily temperature dropped to −7.9°C, and the minimum temperature reached −12.0°C at 22:00 (Figure 2c).

### 3.2 Seasonal Differences in the Spatial Distribution of Bharal

**FIGURE 3:**
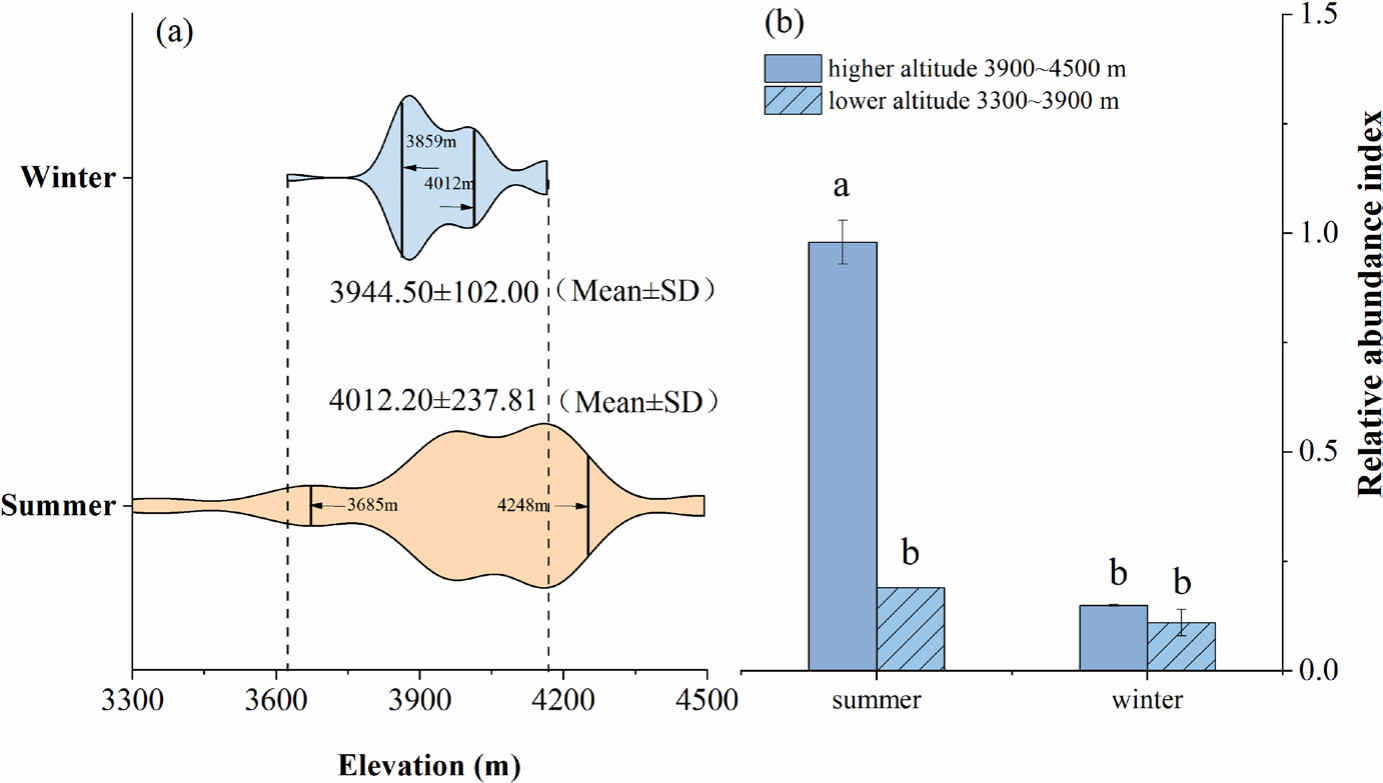
Seasonal differences in the spatial distribution of bharal. (a) The altitude interval of bharal distribution during summer and winter, with the width of the violin plot indicating the RAI of bharal there. (b) The spatial variation in inter‐seasonal activity intensity of bharal. Graphs marked with the same letter indicate non‐significant differences, different letters indicate significant differences (significant level *α* = 0.05). The same applies below.

The Bharal population primarily inhabited the sub‐alpine meadow area above 3300 m in Xuebaoding, exhibiting distinct seasonal variations in spatial distribution. The distribution range of bharal was wider during summer (3300–4495 m) compared to winter (3623–4166 m). Furthermore, during summer, bharal maintained a high activity intensity (RAI > 10) across a broader altitude range (3685–4248 m, with an altitude difference of 563 m). During winter, their activity was limited to a narrower altitude interval (3859–4012 m, with a elevation difference of 153 m) (Figure 3a). Spatial activity characteristics revealed that bharal exhibited significantly higher activity at higher altitude during summer (*p* < 0.05), while no significant spatial difference in activity was observed during winter (Figure 3b).

### 3.3 Seasonal Differences in Activity Rhythm of Bharal

**FIGURE 4:**
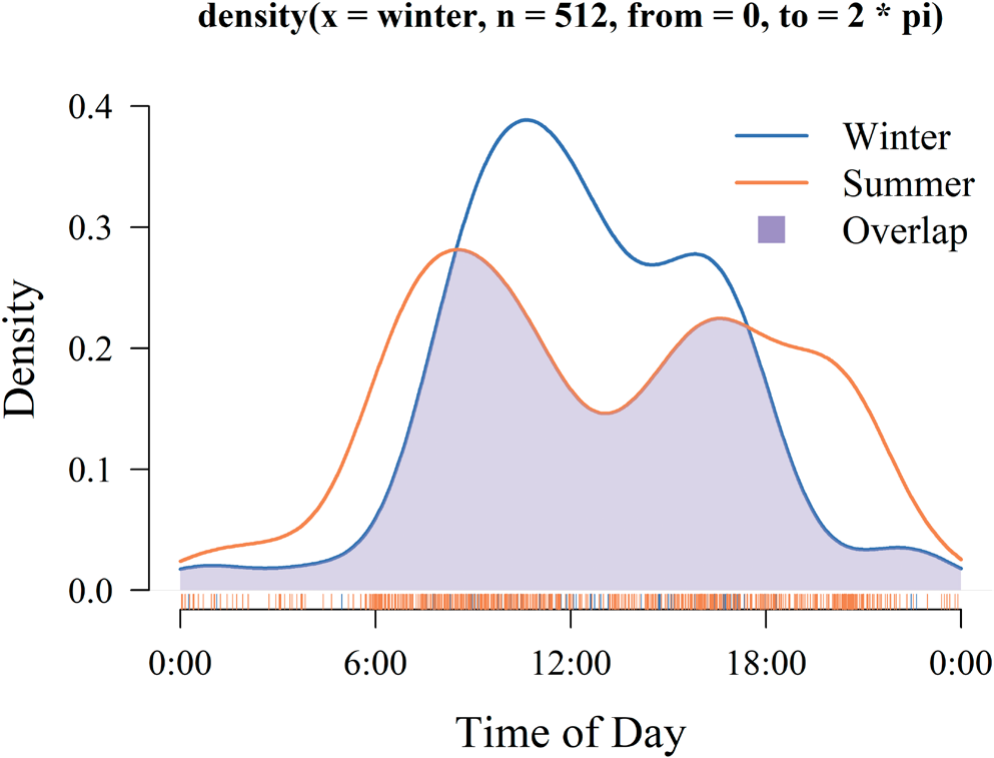
Analysis of daily activity rhythm of bharal during summer and winter. The horizontal axis represents the time of day. The vertical axis represents the probability that the target species was detected at that point in time.

Bharal exhibited typical ungulate activity rhythm patterns, characterized by heightened activity during dawn and dusk (Figure 4). In winter, bharal were frequently captured between 8:00–11:00 and 16:00–17:00, while in summer, they were often captured between 7:00–10:00 and 15:00–17:00. The duration of continuous activity (the interval between activity peaks) was approximately 12 h in summer and about 7 h in winter. Significant differences (*p* < 0.01) were observed between the daily activity rhythm curves of bharal between summer and winter. The difference between the peak activity times in winter and summer was nearly 3 h. The overlap coefficient (Δ_1_) was 0.71. The onset of winter activity peaks occurred approximately 1 h later than in summer. There was a prolonged interval between morning and evening activity peaks in summer.

**FIGURE 5:**
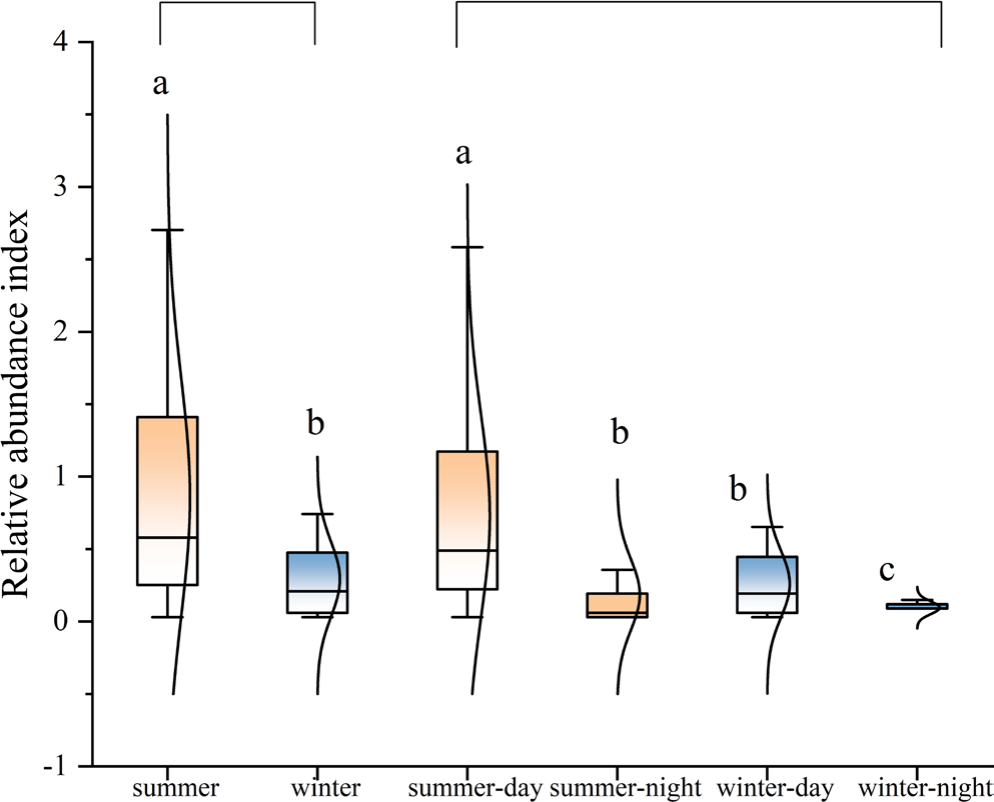
Activity intensity and diurnal differences of bharal during summer and winter.

The activity levels of Bharal were found to be significantly higher during summer (RAI = 0.85 ± 0.04) compared to winter (0.29 ± 0.21, *p* < 0.05) (Figure 5). Diurnal and nocturnal activities exhibited greater prominence during summer (0.73 ± 0.20; 0.19 ± 0.01) than in winter (0.25 ± 0.01, *p* < 0.05; 0.10 ± 0.05, *p* < 0.05). Both daytime and nighttime activity intensities were significantly elevated during summer compared to winter (0.73 ± 0.20; 0.25 ± 0.01) (0.19 ± 0.01, *p* < 0.05; 0.10 ± 0.05, *p* < 0.05).

## 4 Discussion

This study offers a comprehensive analysis of bharal's adaptive strategies by integrating temporal and spatial dimensions, utilizing camera‐trap datasets and NDVI within the Giant Panda National Park. Bharal exhibit a finely tuned synchronization between temporal activity and spatial distribution, reflecting their capacity to adjust time‐activity budgets and habitat use in response to seasonal resource fluctuations.

Firstly, temporal energy banking: the marked reduction in winter activity intensity (RAI 0.29 compared to 0.85, representing a 66% reduction) minimizes thermoregulatory costs while preserving essential rutting behaviors (Jiang and Migmar 2020). This strategy resembles capital breeding tactics observed in Cervidae (Arnold et al. 2004). Bharal exhibit a unique diurnal‐nocturnal modulation (*p* < 0.05). This temporal recalibration likely mitigates thermoregulatory costs under cold conditions (with an average site temperature of −7.9°C), balancing energy conservation with reproductive demands (Chen et al. 2021). Almost 6 h shortening of the daily activity period during winter further illustrated the energy‐saving strategy predominating bharal's seasonal behavior. These activity adjustments align with ungulate strategies like red deer's ( *Cervus elaphus* ) winter reduction in nocturnal activity under a capital‐breeder model (Arnold et al. 2004).

Secondly, spatial metabolic optimization: the winter range contracted by 73%, concentrating between elevations of 3859 and 4012 m, which reduces snow locomotion costs (Hartley et al. 2023). It also ensures access to thermal refugia, conferring a key survival advantage over non‐contracting ungulates (Beumer et al. 2020). This spatial compression closely tracks NDVI variations, reflecting a finely tuned response to fluctuating resource availability. Although NDVI has inherent limitations as an indicator of overall resource abundance and nutritional quality, it remains the most reliable proxy available. The preference for lower elevations in winter can be attributed to diminished snow cover, thereby minimizing locomotion energy expenditure, alongside the benefits of thermal buffering by complex terrain (Milling et al. 2018). These parallel adaptive behaviors are observed in other alpine ungulates, such as muskoxen ( *Ovibos moschatus* ), that reduce winter foraging activity to avoid energy inefficient movement in snow‐covered habitats (Beumer et al. 2020). During summer, bharal expand their altitudinal range upward by 563 m into nutrient‐rich high‐altitude meadows, maximizing energy intake during peak vegetation growth. In contrast, winter prompts a contraction to mid‐elevations, where they trade broader spatial coverage for energy conservation amid harsh environmental conditions. This interplay between activity intensity and spatial dynamics offers a novel perspective for refining climate change models targeting alpine species, as energy balance likely plays a pivotal role in driving their seasonal adaptive responses.

Our study not only documents bharal's sensitivity to seasonal variability but also provides insights into broader alpine ungulate strategies. For instance, extreme activity plasticity mirrors that of muskoxen (Côté et al. 1997) or Himalayan tahr ( *Hemitragus jemlahicus* ) (Green 1978), while altitudinal migration patterns resemble those observed in moose ( *Alces alces* ) (Nordengren 1997; Wehr et al. 2024) and takin (Guan et al. 2013). However, the bharal's unique integration of temporal and spatial strategies reveals a nuanced approach to managing the energy‐cost trade‐offs inherent to alpine ecosystems. The implications for conservation are significant. Bharal in Giant Panda National Park operate within a “sky island”‐like system (Browne and Ferree 2007), effectively isolated above the forest line (3200–4100 m) despite the proximity (< 60 km) to other alpine meadows, such as those in the Tangjiahe Reserve. Habitat fragmentation, driven by livestock grazing and by villages and farmland near valley bottoms (Hartley et al. 2023), is a major barrier to dispersal and further compounds the challenges of climate change adaptation.

The methodological integration of large‐scale camera‐trap monitoring, coupled with satellite‐derived vegetation indices, demonstrates the power of spatiotemporal frameworks for studying adaptation in alpine ecosystems (Pettorelli et al. 2014). These tools provide an empirical foundation for predicting climate‐driven behavioral and distributional shifts, underscoring the urgency of implementing cross‐reserve connectivity corridors and strengthening habitat management in fragmented mountain ecosystems (Bischof et al. 2012; Liang et al. 2021). Finally, by expanding our understanding of bharal adaptation mechanisms, this research contributes both to alpine ecology theory and to the development of conservation strategies tailored to mitigate the dual threats of habitat fragmentation and climate change.

## Author Contributions

**Rumei Zhang:** conceptualization (equal), data curation (lead), formal analysis (lead), investigation (supporting), methodology (equal), software (lead), validation (equal), visualization (lead), writing – original draft (lead), writing – review and editing (equal). **Chen Yang:** conceptualization (equal), formal analysis (equal), investigation (equal), methodology (equal), project administration (equal), resources (equal), supervision (equal), validation (equal), visualization (equal), writing – original draft (supporting), writing – review and editing (equal). **Ding Zhao:** data curation (equal), investigation (lead), project administration (equal), resources (lead), supervision (equal), validation (equal). **Dehong Pang:** data curation (equal), investigation (equal), project administration (equal), resources (lead), supervision (equal), validation (equal). **Weichao Zheng:** investigation (equal), methodology (equal), writing – review and editing (equal). **Tianpei Guan:** conceptualization (equal), data curation (equal), formal analysis (supporting), funding acquisition (lead), investigation (lead), methodology (equal), project administration (lead), resources (lead), software (equal), supervision (lead), validation (lead), visualization (equal), writing – original draft (equal), writing – review and editing (lead). **Zhuo Tang:** conceptualization (equal), data curation (equal), formal analysis (equal), investigation (equal), methodology (equal), resources (equal), supervision (equal), validation (equal), visualization (equal), writing – original draft (equal), writing – review and editing (equal).

## Ethics Statement

Our research was entirely conducted through non‐contact observation.

## Conflicts of Interest

The authors declare no conflicts of interest.

## Supporting information


**Data S1:** Infrared camera footage documenting the activity of bharal, including the specific dates and times of the recorded events.

## Data Availability

Data are available as Supporting Information.
